# *Leucothoe kawesqari*, a new amphipod from Bernardo O’Higgins National Park (Chile), with remarks on the genus in the Magellan Region (Crustacea, Peracarida)

**DOI:** 10.3897/zookeys.539.6157

**Published:** 2015-11-23

**Authors:** Patricia Esquete, Cristian Aldea

**Affiliations:** 1 Departamento de Biologia & CESAM, Universidade de Aveiro, Portugal; 2 Laboratorio de Ecología y Medio Ambiente, Instituto de la Patagonia, Universidad de Magallanes, Punta Arenas, Chile; 3 Programa GAIA-Antártica, Universidad de Magallanes

**Keywords:** Pacific Ocean, channels and fjords, Southern Ocean, benthos, *Macrocystis pyrifera*, Océano Pacífico, Canales y Fiordos, Océano Antártico, bentos, *Macrocystis pyrifera*

## Abstract

Although the genus *Leucothoe* has been reported repeatedly in the Magellan Region, the citations in the Channels and Fjords Ecoregion were either unidentified or attributed to the previously considered cosmopolitan *Leucothoe spinicarpa*. In this work, *Leucothoe kawesqari* **sp. n.** is described, which can be distinguished from other species of the genus in the Southern Ocean by having eyes present, epimeral plates with no setae, anterior coxae not acutely produced or excavate, coxa 5 slightly bilobed, accessory flagellum present, mandibular palp article 3 shorter than ½ article 2, pereopods 5–7 basis expanded, ovoid, posterior margin weakly crenulate and telson apex irregularly truncated. The new species was found in hard substrates, both unvegetated and with macroalgae, mainly in kelp forest of *Macrocystis pyrifera*.

## Abstract (es)

A pesar de que el género *Leucothoe* ha sido citado en la región Magallánica en repetidas ocasiones, las citas en la Ecorregión de Canales y Fiordos o bien no han sido identificados o atribuidos a la antes considerada especie cosmopolita *Leucothoe spinicarpa*. En este trabajo se describe *Leucothoe kawesqari* **sp. n.**, que se distingue de otras especies del género que se encuentran en el Océano Antártico por presentar ojos, placas epimerales sin setas, coxas anteriores no puntiagudas ni excavadas, coxa 5 ligeramente bilobulada, flagelo accesorio, artículo 3 del palpo mandibular más corto que la mitad del artículo 2, bases de los pereiópodos 2–7 expandidas con el margen ligeramente crenulado y ápice del telson truncado e irregular. La nueva especie fue encontrada en sustratos duros, tanto sin vegetación como con macroalgas, dominadas por bosques de huiros de la especie *Macrocystis pyrifera*.

## Introduction

*Leucothoe* Leach, 1814 is a speciose amphipod genus that currently comprises 132 species (WoRMS 2015). Species of *Leucothoe* are widespread in all oceans and inhabit a variety of benthic environments from the intertidal zone to –3570 m although most of them live in shallow waters in association with other invertebrates such as in ascidians, sponges, and bivalve mollusks, or free living in algae or coral rubble (White 2011).

The genus *Leucothoe* has been reported repeatedly in the Magellan Region, defined as the Patagonian shelf south of about 41°S on both the Pacific and Atlantic sides, assigned to the wrongly considered cosmopolitan species *Leucothoe spinicarpa* (Abildgaard, 1789) (see De Broyer et al. 2007, White 2011). Krapp-Schickel and De Broyer (2014) revised the *Leucothoe* in the Southern Ocean, and clarified the citation of Holman and Watling (1983) from Tierra de Fuego by describing the species *Leucothoe weddellensis* Krapp-Schickel & De Broyer, 2014. The rest of the records of the genus in the region do not provide descriptions, hence the specimens cannot be attributed to any described species. In this work, a new species found in the channels and fjords of Bernardo O’Higgins National Park (henceforward BONP) is described.

## Materials and methods

BONP is placed in the Chilean geopolitical regions of Aysén and Magallanes, between 48.0–51.6°S and 73.3–75.8°W (Aravena et al. 2011). Its coastal line consists of countless channels and fjords along more than 400 linear kilometers of the southeastern Pacific (Aldea et al. 2011), which house a variety of habitats.

Between January and March 2010 two cruises were carried out onboard the vessel *MV Nueva Galicia* with the objective of sampling the rocky sublittoral bottoms of the channels and fjords of BONP. A total of 23 sites was sampled with SCUBA: five samples were taken manually at both 5 and 15 m depth at each site (10 samples at each site), harvesting squares of 25×25 cm (0.063 m^2^) by scraping off all the organisms (including fauna and smaller algae), but not the kelps. Samples were fixed in 5% buffered formalin and subsequently sorted, preserved in 70% alcohol and identified. Illustrations were performed using a *camera lucida* connected to a compound microscope.

Terminology used in the description follows Krapp-Schickel and De Broyer (2014). Body length is measured from dorsodistal extreme of pereon to frontal tip of head. Specimens with no penile process or marsupium are considered neuters. Type material is lodged in the collections of the Museo Nacional de Historia Natural, Chile (MNHNCL) and the Museo Nacional de Ciencias Naturales de Madrid (MNCN), Spain.

## Results

### Systematics Order Amphipoda Latreille, 1816 Suborder Gammaridea Latreille, 1802 Family Leucothoidae Dana, 1852 Genus *Leucothoe* Leach, 1814

#### Leucothoe kawesqari sp. n.

Taxon classificationAnimaliaAmphipodaLeucothoidae

http://zoobank.org/B87DCE33-A922-4252-96AD-478362B416B1

##### Material examined.

Holotype: female, 8 mm length, MNHNCL AMP-15038; Paratypes: female, dissected, 5 mm length, MNHNCL AMP-15039; 2 females, MNCN 20.04/10146, MNCN 20.04/10147; All, 51°04'04.7"S, 74°08'29.5"W, 5–15 m depth, rocks, 27 January 2010. Other material examined: 8 females and neuters, 51°04'04.7"S, 74°08'29.5"W, 5–15 m depth, rocks, 27 January 2010; 16 neuters, 49°36'16.7"S, 75°23'31.4"W, 5–15 m depth, rocks with macroalgae, 19 March 2010, 1 female, 49°11'27.1"S, 75°23'30.8"W, 5–15 m depth, rocks with macroalgae, 19 March 2010. All coll. R. Barría, E. Newcombe, M. Hüne and T. Césped.

##### Diagnosis.

Head anterior margin rounded, mid-cephalic keel quadrate but not prominent. Eyes present. Epimeral plates with no setae, epimeron 3 posterior margin excavate, postero-ventral corner blunt, with right angle. Coxae 1-3 not acutely produced, nor excavated, coxa 3 longer than broad, coxa 5 slightly bilobed. Antenna 1 main flagellum of 11–12 articles, accessory flagellum present, minute, of two unequal segments. Antenna 2 flagellum of 6 articles. Mandibular palp article 3 shorter than ½ of article 2. Ganthopod 1 propodus palm with minute serrations. Dactylus reaching 0.4 of propodus length. Gnathopod 2 basis posterior margin smooth, carpus smooth, without tooth or process, setose, reaching 0.3 of propodus length, propodus with short, blunt distal prolongation and three medial rows of setae. Pereopods 5–7 bases expanded, ovoid, posterior margin weakly crenulate. Telson apex irregularly truncated.

##### Derivatio nominis.

Named after the Alacalufe people Kawésqar, whose ancestral territory extends through the Magellan Region, from the Gulf of Penas to the Strait of Magellan.

**Description.**

**Figure 1.**
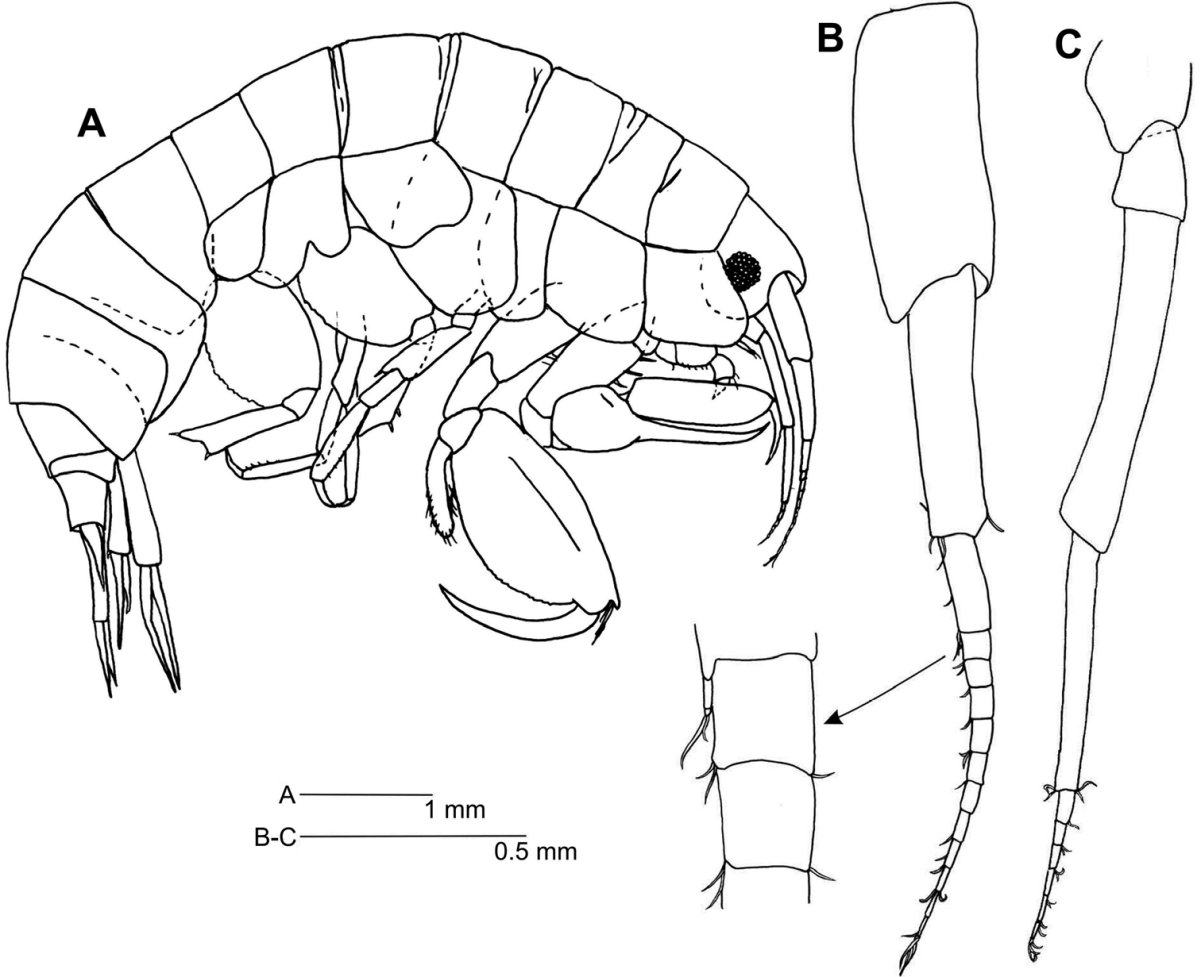
*Leucothoe kawesqari* sp. n. **A** Habitus **B** Antenna 1 **C** Antenna 2.

*Body* (Fig. 1A) length 5–8 mm.

*Head* (Fig. 1A) anterior margin rounded, anterodistal margin rounded. Mid cephalic keel quadrate but not prominent, eyes present, rounded.

*Antenna 1* (Fig. 1B) 0.4 times as long as body; peduncle article 1 2.3 times as long as broad; article 2 subequal in length, 4.3 times as long as broad; article 3 half as long as long article 2, 2.8 times as long as broad; accessory flagellum present, minute, about ½ as long as main flagellum article 1, biarticulated, first article about three times as long as second; main flagellum of 11 articles, about as long as peduncle article 1, aesthetascs present, flagellum article 1 as long as articles 2–3 and half of 4 together.

*Antenna 2* (Fig. 1B) slightly shorter than antenna 1; peduncular article 5 0.8 times as long as article 4; flagellum of 6 articles.

**Figure 2.**
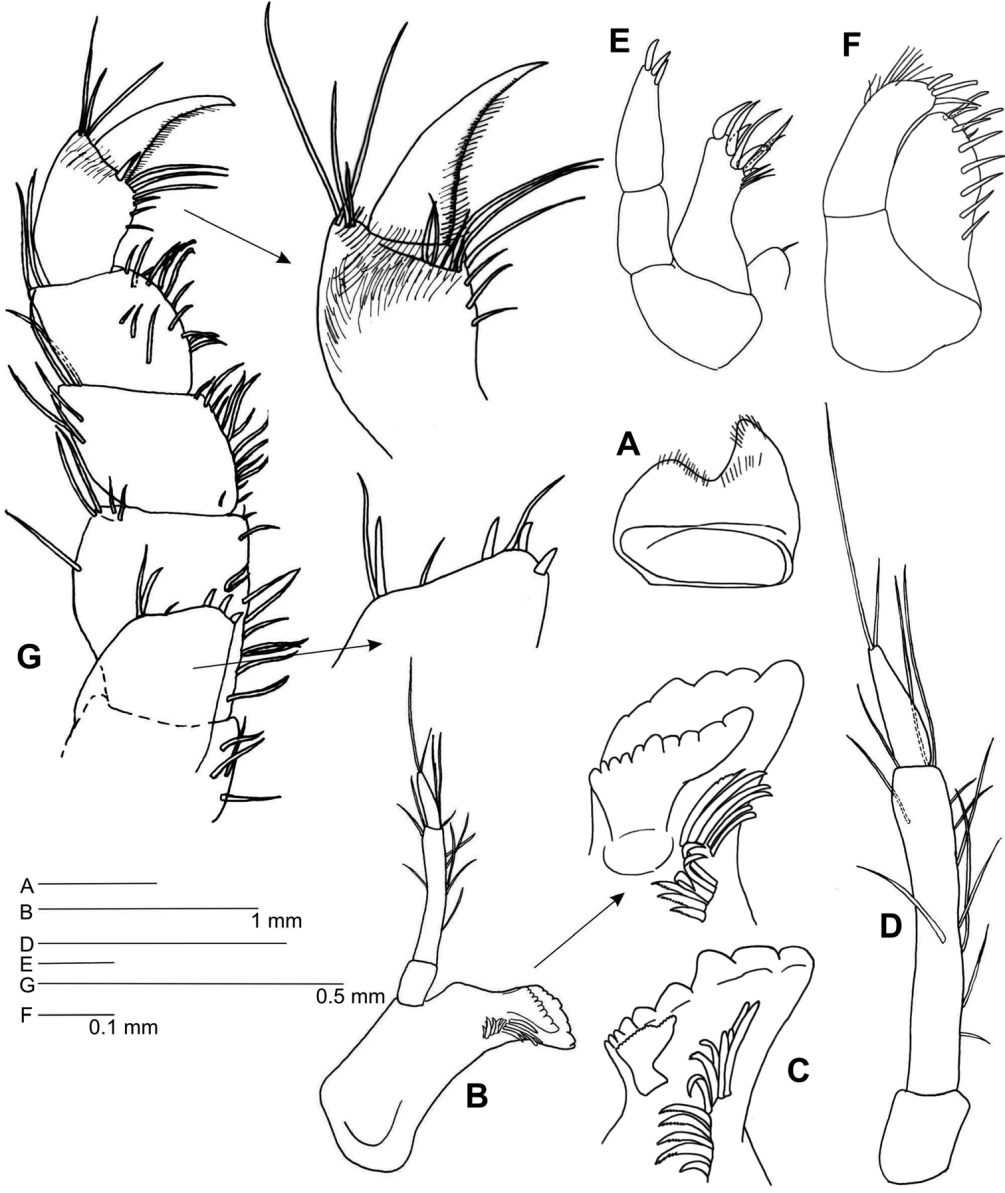
*Leucothoe kawesqari* sp. n. **A** Upper lip **B** Left mandible **C** Right mandible **D** Mandibular palp **E** Maxilla 1 **F** Maxilla 2 **G** Maxilliped.

*Mouthparts*. Upper lip (Fig. 2A) asymmetrically lobate, anterior margin setose. Mandibles (Fig. 2B, C) lacking molars; mandibular palp (Fig. 2D) article 2 with 9 lateral and 3 distal setae, article 3 about 1/3 of article 2, with two unequal distal setae; incisor dentate, spine row of 12 serrate spines; left lacinia mobilis (Fig. 2B) large, distally as long as incisor; right lacinia mobilis (Fig. 2C) small, weakly dentate, Maxilla 1 (Fig. 2E) palp two-articulate, distal article with three distal spines; outer plate with four distal spines, three distal slender spines and three subdistal fine setae; inner plate with one distal small seta. Maxilla 2 (Fig. 2F) outer plate with three distal spines, outer margin subdistally setulose; inner plate with nine spines along inner margin. Maxilliped (Fig. 2G) inner plate distal margin with three setae, three short spines on inner corner and one slender spine on outer corner; palp articles 2–4 similar in length.

**Figure 3.**
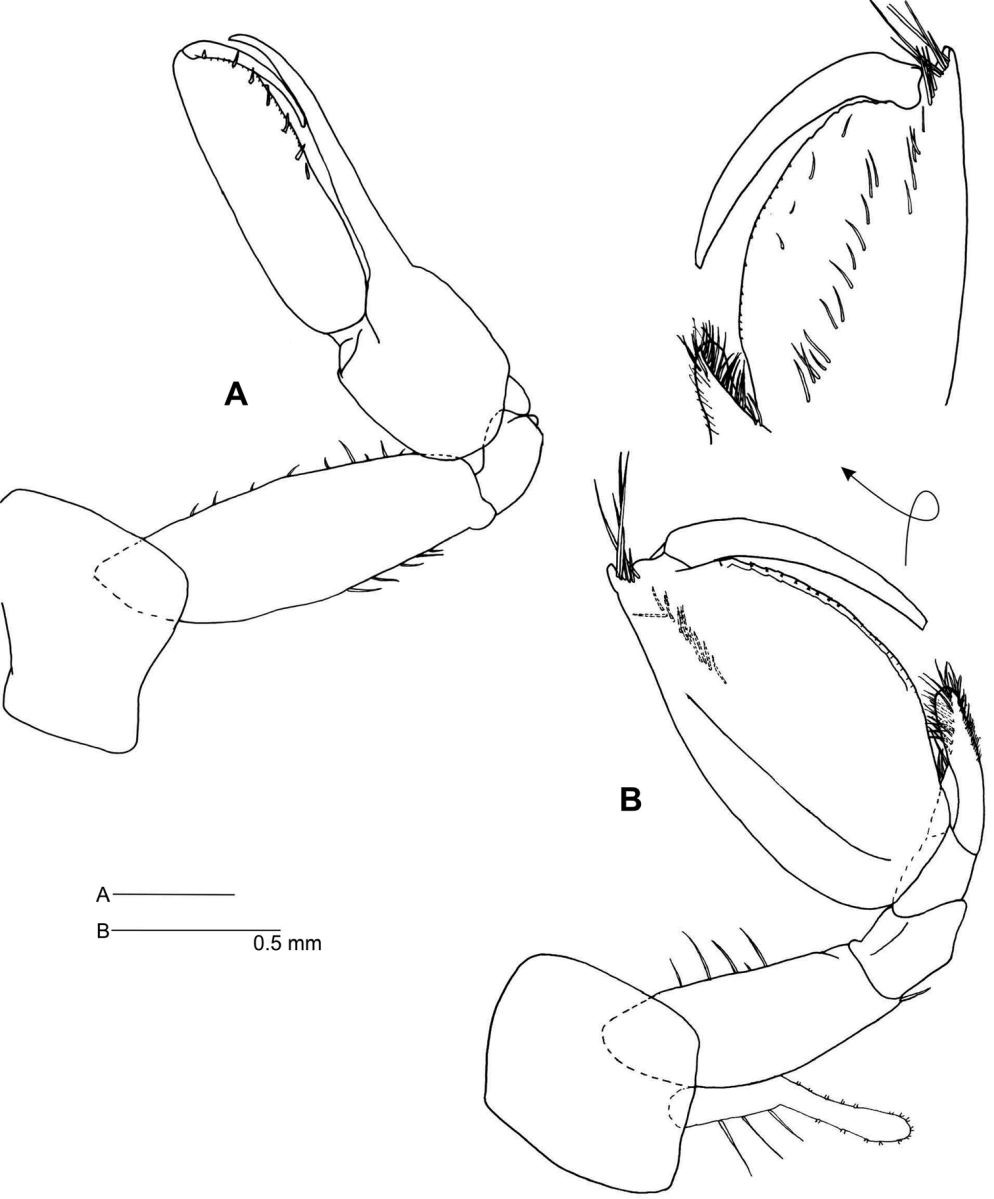
*Leucothoe kawesqari* sp. n. **A** Ganthopod 1 **B** Gnathopod 2.

*Gnathopod 1* (Fig. 3A) coxa naked, anterior margin 1.4 as long as posterior; basis anterior and posterior margins with sparse short setae; ischium naked; carpus linear, naked; propodus 3.6 times as long as broad, palm with minute serrations and row of 7 short setae; dactylus smooth, reaching 0.4 length of propodus.

*Gnathopod 2* (Fig. 3B) coxa subquadrate, naked; basis anterior margin with 4–5 setae, posterodistal corner with one seta; carpus reaching 0.3 of propodus length, curved, distally rounded, densely setose; propodus twice as long as broad, anterodistal margin with short, blunt prolongation bearing a tuft of setae, with three facial rows of setae, osp. n.rse and near palm, one mediofacial and reaching 2/3 the length of propodus, and one displaced dorsally, reaching from distal corner to 1/3 length of propodus, palm convex, slightly crenulated; dactylus smooth, reaching 0.6 length of propodus.

**Figure 4.**
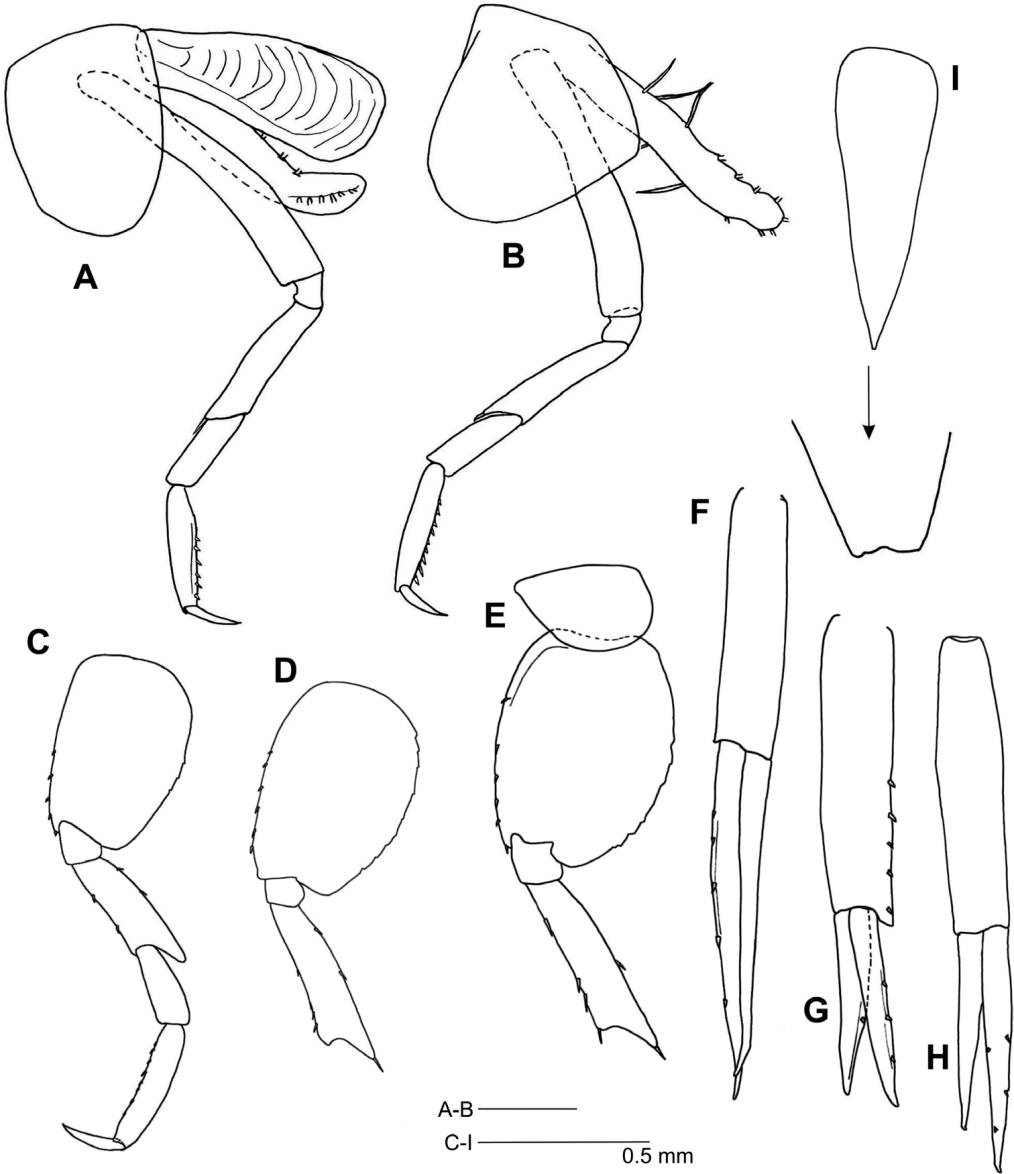
*Leucothoe kawesqari* sp. n. **A** Pereopod 3 **B** Pereopod 4 **C** Pereopod 5 **D** Pereopod 6 **E** pereopod 7 **F** Uropod 1 **G** Uropod 2 **H** Uropod 3 **I** Telson.

*Pereopod 3* (Fig. 4A) coxa longer than broad, distal margin rounded, naked; basis very narrow, naked; merus with anterodistal spine; propodus with a row of six ventral, short spines.

*Pereopod 4* (Fig. 4B) coxa naked, anterior margin longer than posterior, distal margin rounded and oblique, posterior margin tapered; propodus with a row of nine ventral, short spines; otherwise as pereopod 3.

*Pereopod 5* (Fig. 4C) coxa naked, slightly bilobed; basis oval, 1.5 times as long as broad, anterior margin with spines, posterior margin weakly crenulated; merus anterior and posterior margins with spines, posterodistal corner lengthened; propodus anterior margin with a row of spinules.

*Pereopod 6* (Fig. 4D) coxa bilobed; basis 1.4 times as long as broad; otherwise as pereopod 5.

*Pereopod 7* (Fig. 4E) coxa shorter than broad, distal margin rounded; otherwise as pereopod 6.

*Epimeral plates* (Fig. 1A) naked; epimeron 2 posterior margin concave, posteroventral corner without cusp; epimeron 3 posterior margin excavate, postero-ventral corner blunt, with right angle.

*Uropods.* Uropod 1 (Fig. 4F) peduncle 0.7 times as long as outer ramus; outer ramus with 5 spines; rami with marginal spines, inner ramus slightly shorter than outer. Uropod 2 (Fig. 4G) somewhat shorter than uropod 1, peduncle 1.6 times as long as outer ramus, with spines on distal half of outer margin; rami with marginal spines, inner ramus slightly shorter. Uropod 3 (Fig. 4H) 1.1 times as long as uropod 1, peduncle 1.2 times as long as outer ramus; outer ramus with marginal spines; the shorter one 0.8 times as long as the longer one, naked.

*Telson* (Fig. 4I) 3.3 times as long as broad, distal tip minutely, irregularly truncate.

##### Remarks.

As mentioned above, the only described species of *Leucothoe* found in the Magellan Region is *Leucothoe weddellensis*. Following Holman and Watling (1983), Krapp-Schickel and De Broyer (2014) divided the material of *Leucothoe weddellensis* in two groups, according to morphological differences, locations and size. The material from the Magellan Region (South of Tierra de Fuego) corresponds to the larger specimens (more than 14 mm long). From those, *Leucothoe kawesqari* differs (besides the length) in that the former lacks accessory flagellum, has a distinctively more slender gnathopod 1 propodus, coxa 5 is markedly bilobed, peropods 5–7 basis are pear-shaped oval (while in *Leucothoe kawesqari* are regularly oval) and more slender, the epimeron 1 is posteriorly serrate, the epimeron 2 has ventrodistal setae, and epimeron 3 as a posterodistal small prolongation. The smaller specimens differ in lacking accessory flagellum, having a longer mandibular palp article 3 (1/2 of the length of article 2), gnathopod 1 propodus anterior margin concave, gnathopod 2 basis more setose, pereopods 5–7 distinctly narrower, pereopods 5–6 with slightly concave hind margin.

*Leucothoe kawesqari* is most similar to *Leucothoe antarctica* Pfeffer, 1888 as redescribed by Krapp-Schickel and De Broyer (2014): they share a mandibular palp article 3 1/3 length of article 2, coxa 3 longer than broad with rounded distal margin, and 5–7 basis oval, but *Leucothoe antarctica* lacks accessory flagellum, has a coxa 5 distinctively bilobed, maxilliped palp article 4 and 5 more slender, setae on gnathopod 2 ischium and merus, pereopod 6 basis strongly serrated, epimeron 2–3 with ventrodistal setae, uropods more spinose and telson with a pair of distal setae.

Regarding other species from the Southern Ocean, *Leucothoe merletta* Krapp-Schickel & De Broyer, 2014 can be readily differentiated from *Leucothoe kawesqari* because of having coxae 2 and 4 with acute anterodistal angles, having mandibular palp article 3 about as long as article 2, pereopods 5–7 basis with regularly rounded hind margin, epimeron 1 distal margin rounded and epimeron 3 with rectangular posterodistal corner. *Leucothoe campbelli* Krapp-Schickel & De Broyer, 2014 has a longer mandibular palp article 3 (1/2 length of article 2), coxa 3 subtrapezoidal, gnathopod 1 propodus more robust, pereopods 5–7 basis posterior margin smoth and epimeral plate with posterodistally upturned corner. *Leucothoe longimembris* Krapp-Schickel & De Broyer, 2014 lacks eyes, no accessory flagellum, mandibular palp article 3 1/2 length of article 2, and basis of pereopods 5–7 slim, broadest proximally. *Leucothoe macquariae* Krapp-Schickel & De Broyer, 2014 lacks accessory flagellum, mandibular palp article 3 1/2 length of article 2, more robust gnathopods carpi, epimeron 3 distal posterior margin distal corner blunt and upturned, and telson tip acute. *Leucothoe orkneyi* Holman & Watling, 1983 can be immediately differentiated in having a very slender gnathopod 1 propodus, and having a very prominent mid-cephalic keel, no accessory flagellum, a very slender, pereopods 5–7 basis margin strongly serrated, and epimera distal margins with setae.

*Leucothoe tolkieni* Vinogradov, 1990 is the only other species described from the Southeastern Pacific, although it was found well offshore. It differs from the species described here mainly in having the head anterior margin truncate with eyes that cover most of the head, gnathopod 1 basis anteroproximally expanded, and propodus curved, proximally inflated, gnathopod 2 carpus distally truncate, spoon-like, pereopods 5–7 bases narrowly expanded and telson apex rounded.

Although previous reports of *Leucothoe spinicarpa* in the Magellan Region are probably wrong (De Broyer et al. 2007, White 2011), it is worth mentioning the main differences with the present species. Based on the description provided by Crowe (2006), unlike *Leucothoe kawesqari*, *Leucothoe spinicarpa* has a gnathopod 1 propodus ventral margin with more than 10 spines, coxa 5 markedly bilobed, gnathopod 2 carpus scarcely setose with a subdistal cusp, epimeron 1 with anterodistal tuft of setae, and telson apex bidentate with a pair of distal setae.

##### Ecology.

*Leucothoe kawesqari* was one of the dominant species of amphipod found in unvegetated hard substrates in the southernmost sampling site, where the amphipods *Polycheria antarctica* (Stebbing, 1875) and *Orchestia* spp., were also abundant. Towards the north of BONP, *Leucothoe kawesqari* was found in subtrates dominated by kelp forest of *Macrocystis pyrifera*, where *Andaniopsis integripes* (Bellan-Santini & Ledoyer, 1986) was dominant and it also co-occurred with the tanaid *Zeuxoides troncosoi* Esquete & Bamber, 2012 and juveniles of the isopod family Janiridae. High abundances of other benthic taxa were found co-occurring with *Leucothoe kawesqari*: the polychaetes *Platynereis australis* (Schmarda, 1861) and *Perinereis gualpensis* Jeldes, 1963, the bivalve *Aulacomya atra* (Molina, 1782) the decapod *Halicarcinus planatus* (Fabricius, 1775) and unidentified species of Echinodermata (Ophiuroidea and Psolidae), Porifera and Ascidiacea. These specimens of *Leucothoe kawesqari* were likely associated with or endocommensal associates of the Porifera and Ascidacea specimens withinin the sample, since the sampling method (scraping substrate) dislodges the samples and everything was sorted through at one time (White 2011, White and Reimer 2012).

## Discussion

**Figure 5.**
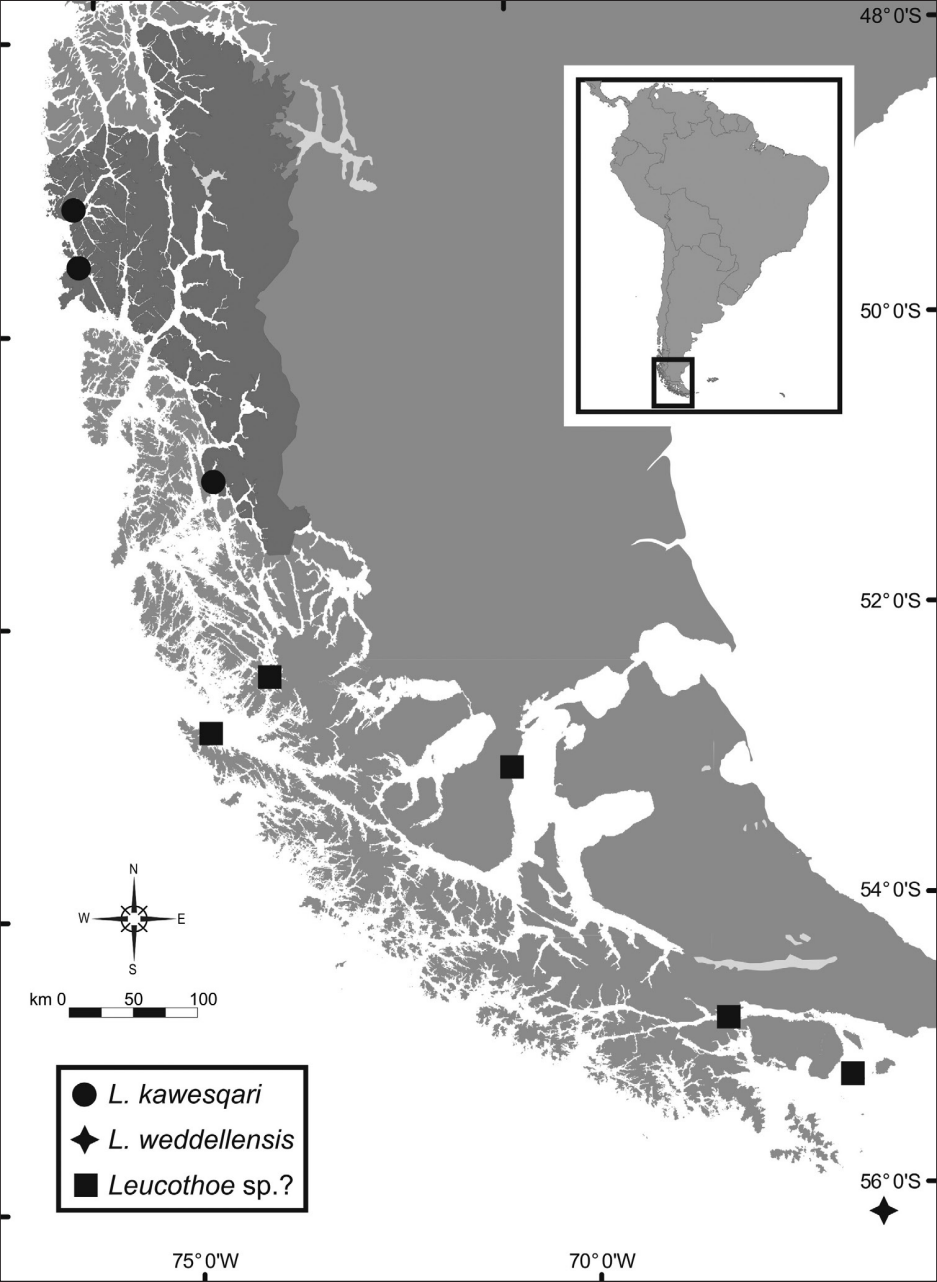
Location of the records of species of *Leucothoe* in the Magellan Region. BONP is shown overshadowed. *Leucothoe* sp.? corresponds to those in Schellemberg (1931) as *Leucothoe spinicarpa* which cannot be attributed to any known species (see text).

The two species currently described for the Magellan Region have a well separated geographical distribution (Fig. 5): While the specimens of *Leucothoe kawesqari* come from the channels and fjords, *Leucothoe weddellensis* was found off shore, south of Tierra de Fuego and is distributed throughout the Antarctic seas (Krapp-Schickel and De Broyer 2014). Schellenberg (1931) reported *Leucothoe* in Cabo Valentina, Rio Seco, Strait of Magellan, Canal Beagle, and Isla Lennox. As mentioned above no illustration was provided, hence his records cannot be attributed to any described species. These locations lie between the distribution areas of the two known species. Otherwise, there are no more records of *Leucothoe* for the southeast coast of the Pacific. Further north, *Leucothoe panpulco* Barnard, 1961 is found in Acapulco and Panamá, and *Leucothoe alata* (Barnard, 1959) in California, with no overlap of distribution ranges of species of *Leucothoe* along the Pacific.

The geographical distribution of the species of *Leucothoe* studied by Krapp-Schickel and De Broyer (2014) and the data presented herein thus complete a latitudinal turnover of *Leucothoe* species along the west coast of the American continent, having from the north toward south, *Leucothoe kawesqari*, *L.* sp. and *Leucothoe weddellensis*. Nevertheless, large areas remain largely undersampled; future surveys in the East pacific including the Magellan region would reveal whether there are regions where species of the genus overlap, or a total latitudinal species turnover due to speciation through colonization.

## Supplementary Material

XML Treatment for Leucothoe kawesqari
